# Design of ultrasensitive Ag-LaFeO_3_ methanol gas sensor based on quasi molecular imprinting technology

**DOI:** 10.1038/s41598-018-32113-x

**Published:** 2018-09-21

**Authors:** Qian Rong, Yumin Zhang, Jicu Hu, Kejin Li, Huapeng Wang, Mingpeng Chen, Tianping Lv, Zhongqi Zhu, Jin Zhang, Qingju Liu

**Affiliations:** School of Physics and Astronomy, School of Materials Science and Engineering, Yunnan Key Laboratory for Micro/nano Materials & Technology, Yunnan University, 650091 Kunming, China

## Abstract

An ultrasensitive methanol gas sensing device based on the quasi-molecular imprinting technology (quasi-MIT) is studied in this work. We applied the sol-gel method (ALS denotes Ag-LaFeO_3_ prepared by the sol-gel method) and combustion synthesis (ALC denotes Ag-LaFeO_3_ prepared by combustion synthesis) to prepare Ag-LaFeO_3_ based sensors. The morphologies and structures of the Ag-LaFeO_3_ materials were examined via various detection techniques. The ALSM and ALCM sensor (ALSM and ALCM denotes the devices prepared by coating the ALS and ALC materials with methanol, respectively) fabricated using the sol-gel method and combustion synthesis combined with quasi-MIT exhibit good gas sensing properties to methanol, in contrast with the two devices (ALSW and ALCW denote the devices prepared for coating the ALS and ALC materials with water, respectively) without the use of quasi-MIT. The results show that quasi-MIT introduced the target gas in the fabrication process of the device, playing an important role in the design of the ultrasensitive methanol gas sensor. The sensing response and the optimum working temperature of ALSM and ALCM gas sensor are 52.29 and 155 °C and 34.89 and 155 °C, respectively, for 5 ppm methanol, and the highest response to other gases is 8. The ALSM and ALCM gas sensors reveal good selectivity and response for methanol.

## Introduction

Methanol is widely used in many fields such as pigments, pharmaceuticals and chemical products. However, it is toxic and causes human nerve poisoning and cardiovascular diseases^1^. Therefore, the preparation of a high response and high selectivity methanol gas sensor has become an urgent problem. Currently, several methods are used to for the gas sensing and detection of methanol such as chromatography^2^, the spectrophotometric method^3^, the electrochemical method^4^, catalytic luminescence^5^ and the gas sensor method^6^. The first four methods require expensive instruments, leading to their high cost and large required volume and making it difficult to apply them widely. Because of its high sensitivity, simple operation, low cost and small device, gas sensing is an effective method for detecting methanol gas. However, current methanol gas sensors cannot be applied in practical use due to low response and poor selectivity^7–9^.

Metal oxide semiconductors have been used in many fields such as photocatalysis^10,11^, solar cells^12^ and as gas-sensitive materials^13^. Among all kinds of gas-sensitive materials, p-type semiconductor LaFeO_3_ is a potential gas-sensitive material due to its high gas sensing properties^14^ and thermostability^15^. However, the response and selectivity of pure LaFeO_3_ is poor. In our previous work^16^, it was shown that the gas sensing properties of LaFeO_3_ can be improved by doping Ag, but for practical use, the response, selectivity and operating temperature need to be improved further.

Therefore, we introduce the quasi molecular imprinting technique (quasi-MIT), which introduces the target gas into the process of material synthesis or device preparation to obtain a porous structure that is for the adsorption and desorption of methanol gas^17^. Additionally, quasi-MIT has the same effect as MIT but is much simpler because it does not require the identification and use of the functional monomer. Hence, we designed the Ag-LaFeO_3_ for ultrasensitive methanol gas sensors based on the quasi-MIT. The mesoporous materials are obtained by the sol-gel method (ALS) and combustion synthesis (ALC). The sensors were fabricated respectively using mixed pure water (ALSW) as well as methanol (ALSM) with the prepared ALS powders during the sensor fabrication process. The meaning of each abbreviation of this report is shown in Table 1. Similarly, ALCW and ALCM sensors were prepared via mixed ALC respectively with the pure deionized water and methanol. The gas-sensitive characteristics and related mechanisms of methanol gas detection by the ALSW, ALSM, ALCW and ALCM were carefully investigated. It was found that ALSM and ALCM exhibited ultrahigh sensitivity.

**Table 1:** Meaning of each abbreviation.

Abbreviation	Role	Preparation method	Solvent
ALS	Ag-LaFeO_3_ gas-sensing materials	sol-gel	/
ALSW	Ag-LaFeO_3_ sensors	sol-gel	water
ALSM	Ag-LaFeO_3_ sensors	sol-gel	methanol
ALC	Ag-LaFeO_3_ gas-sensing materials	combustion synthesis	/
ALCW	Ag-LaFeO_3_ sensors	combustion synthesis	water
ALCM	Ag-LaFeO_3_ sensors	combustion synthesis	methanol

**Figure 1:**
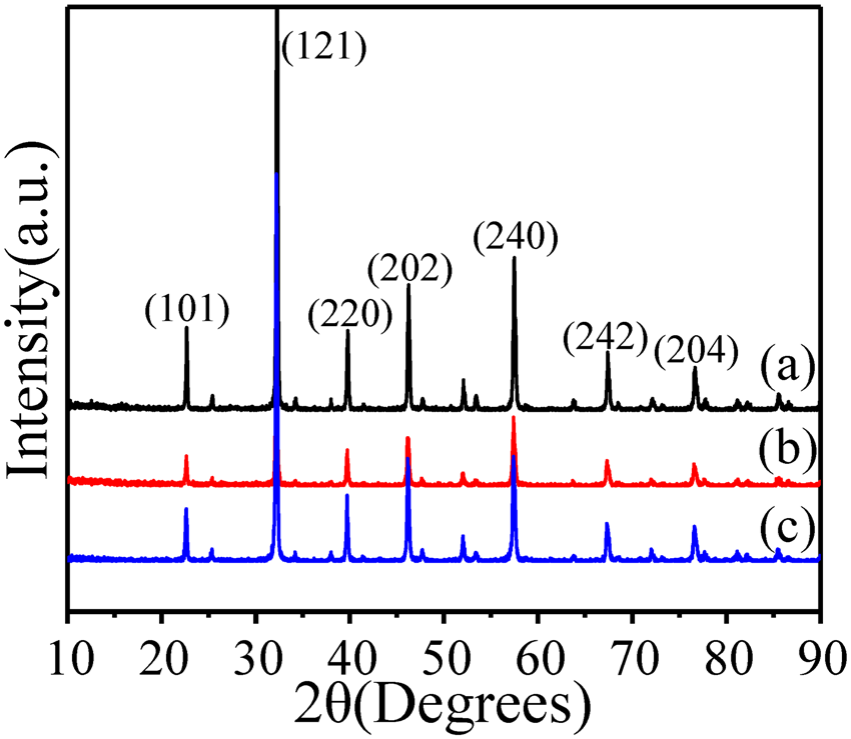
XRD patterns of (**a**) Ag-LaFeO_3_ precursor, (**b**) ALS, and (**c**) ALC.

After the pre-synthesized Ag-LaFeO_3_ precursor was obtained, ALS and ALC were sintered in 800 °C to 2 h in the air, with the XRD results showing the crystalline nature of the sample. All peaks are completely identical with the orthorhombic structure of LaFeO_3_ as shown in Fig. 1. This diffraction pattern perfectly matches the standard JCPDS card no. 37–1493^18^. No precursor residue was detected, indicating that the samples are highly pure. The amount of Ag is so small (mol (Ag): mol (Ag-LaFeO_3_ precursor) = 1%) that it cannot be detected by XRD and FTIR. The lattice parameters obtained from the refinement of the PXRD data are presented in Table 2. It is noteworthy that even though the substitution of Ag does not change the orthorhombic symmetry of the material, it results in a pseudo-tetragonal unit cell with the cell parameter ‘a’ almost equal to ‘c’. It can also be seen from the data presented in Table 2 that the lattice parameters of ALS and ALC show a slight change compared to other reports in the literature^19–26^.

**Table 2:** Lattice parameters of synthesized LaFeO_3_ and their comparison with values reported in the literature.

Lattice parameters	a (Å)	b (Å)	c (Å)	α (°C)	β (°C)	λ (°C)	References
Materials							
Standard LaFeO_3_	5.566	7.854	5.553	90	90	90	/
ALS	5.554	7.851	5.545	90	90	90	This work
ALC	5.561	7.852	5.557	90	90	90	This work
LaFeO_3_	5.562	7.846	5.556	90	90	90	^19^
LaFeO_3_	5.556	7.844	5.555	90	90	90	^20^
LaFeO_3_	5.562	7.852	5.538	90	90	90	^21^
LaFeO_3_	5.562	7.850	5.553	90	90	90	^22^
LaFeO_3_	5.562	7.856	5.558	90	90	90	^23^
LaFeO_3_	5.561	7.858	5.558	90	90	90	^24^
LaFeO_3_	5.558	7.851	5.542	90	90	90	^25^
LaFeO_3_	5.561	7.853	5.568	90	90	90	^26^

**Figure 2:**
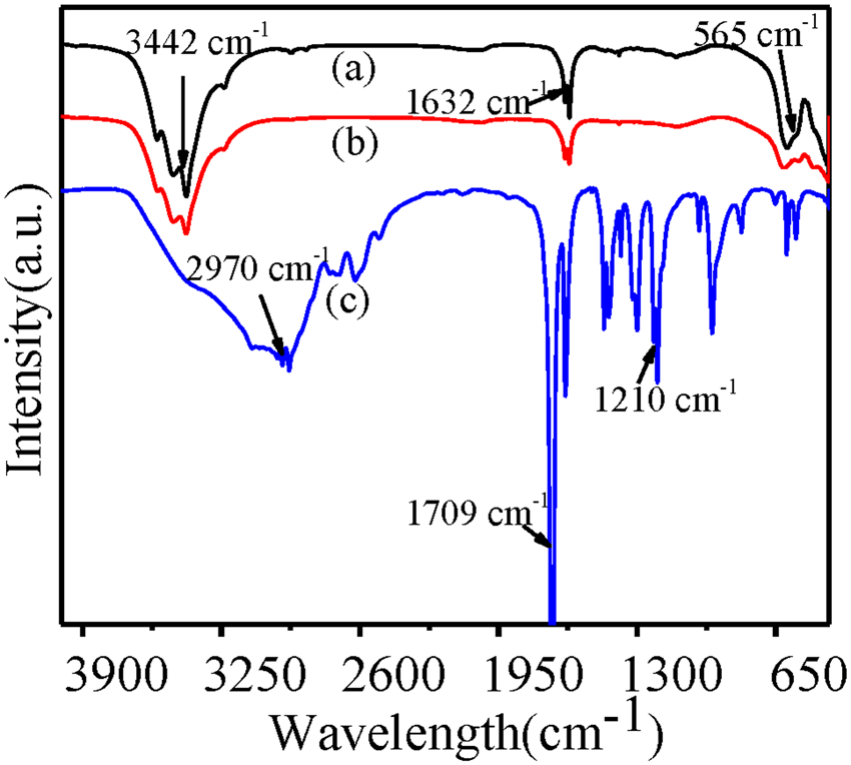
FT-IR spectrum of (**a**) Ag-LaFeO_3_ crosslinker, (**b**) ALS and (**c**) MAA.

To check the purity of the samples and the interaction between the functional groups, we analyzed the Ag-LaFeO_3_ cross-linker, ALS and methacrylic acid (MAA) by FT-IR spectroscopy in the 500–4000 cm^−1^ range as shown in Fig. 2. The FT-IR spectrum of the synthesized Ag-LaFeO_3_ cross-linker (curve (a)) exhibits a peak at ~3442 cm^−1^ corresponding to the stretching vibration of -OH of H_2_O^27^. The strong peaks at ~565 cm^−1^ and ~1632 cm^−1^ are the flexural vibration of Fe-O and La-O. Curve (c) shows the curve of MAA, and the peak around ~1210 cm^−1^ and the peak at ~1709 cm^−1^ are the vibration of C-O and carbonyl in carboxylic acid, respectively. Curve (b) shows the curve of ALS, where the disappearance of the peaks at ~1210 cm^−1^ and ~1709 cm^−1^ compared to the MAA curve suggested an interaction between Ag-LaFeO_3_ and MAA. The mutual effect is attributed to the coordination between the groups in MAA and lanthanum in the Ag-LaFeO_3_ cross-linker, and it is proven that the metal carbonyl complex is formed^28^.

**Figure 3:**
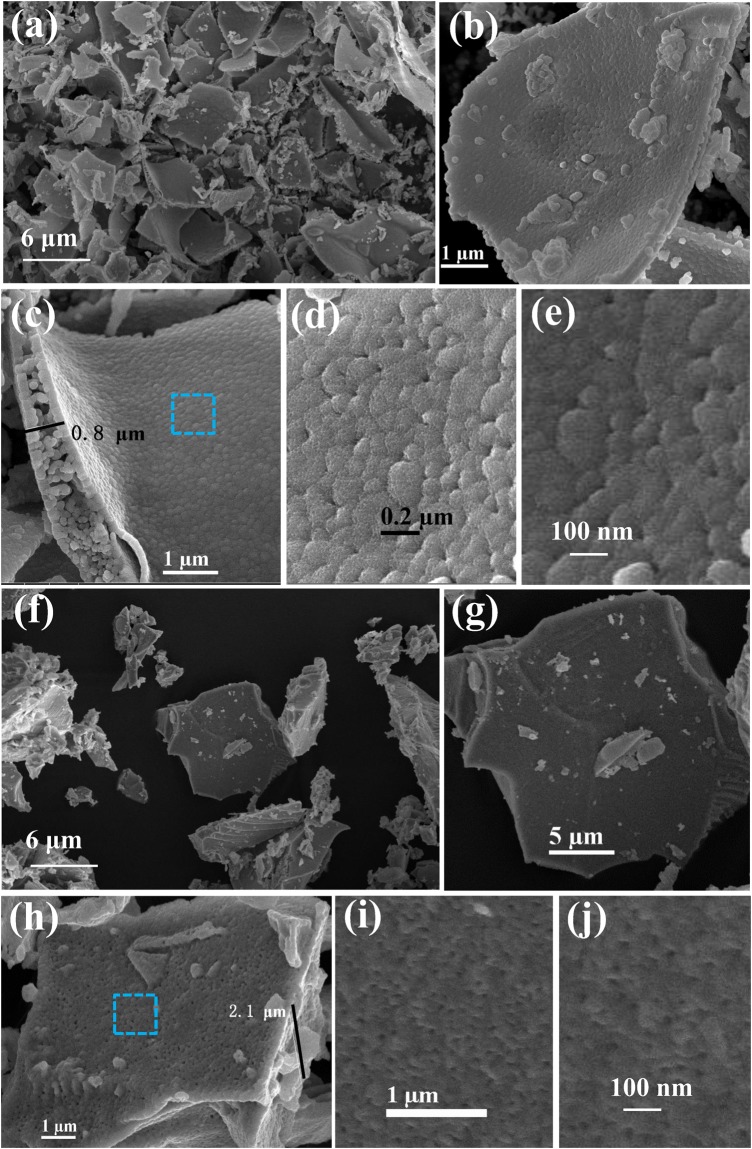
SEM images of as-synthesized ALC and ALS, (**a**) low-magnification image of ALC (5000x), (**b**) plane of ALC (40000x), (**c**) cross-section of ALC (50000x), (**d**) amplification of selected area in (**c**,**e**) high-magnification image of ALC (300000x), (**f**) low-magnification image of ALS (5000x), (**g**) plane of ALS (20000x), (**h**) cross-section of ALS (50000x), (**i**) amplification of selected area in (**h**, **j**) high-magnification image of ALS (400000x).

The pattern and microspore structure of the ALC and ALS were detected via SEM and are shown in Fig. 3. Fig. 3(a,b) shows the appearance of the ALC as a tile, and Fig. 3(f,g) (at low magnification) exhibits stone-like morphologies of the ALS. Fig. 3(c,h) shows featured outer-sides of the ALC and ALS. The ALC shows bulk with porous features composed of many interconnected smaller sized particles, and the thickness of this sample is 0.8 μm. The outside of ALS is solid with the thicknesses of 2.1 μm. The enlarged image of the selected areas of the ALC and ALS samples (Fig. 3(d,i)) shows a porous structure with a micron-scale size. The number of the pores on the surface of ALS is greater than that on the ALC surface. High-powered SEM images in Fig. 3(e,j) are also clearly displayed. Porous morphologies of the samples would make the target gases diffuse in or escape from the inner parts of the sensing films faster than for their solid counterparts and consequently will improve the responses to the target gases.

**Figure 4:**
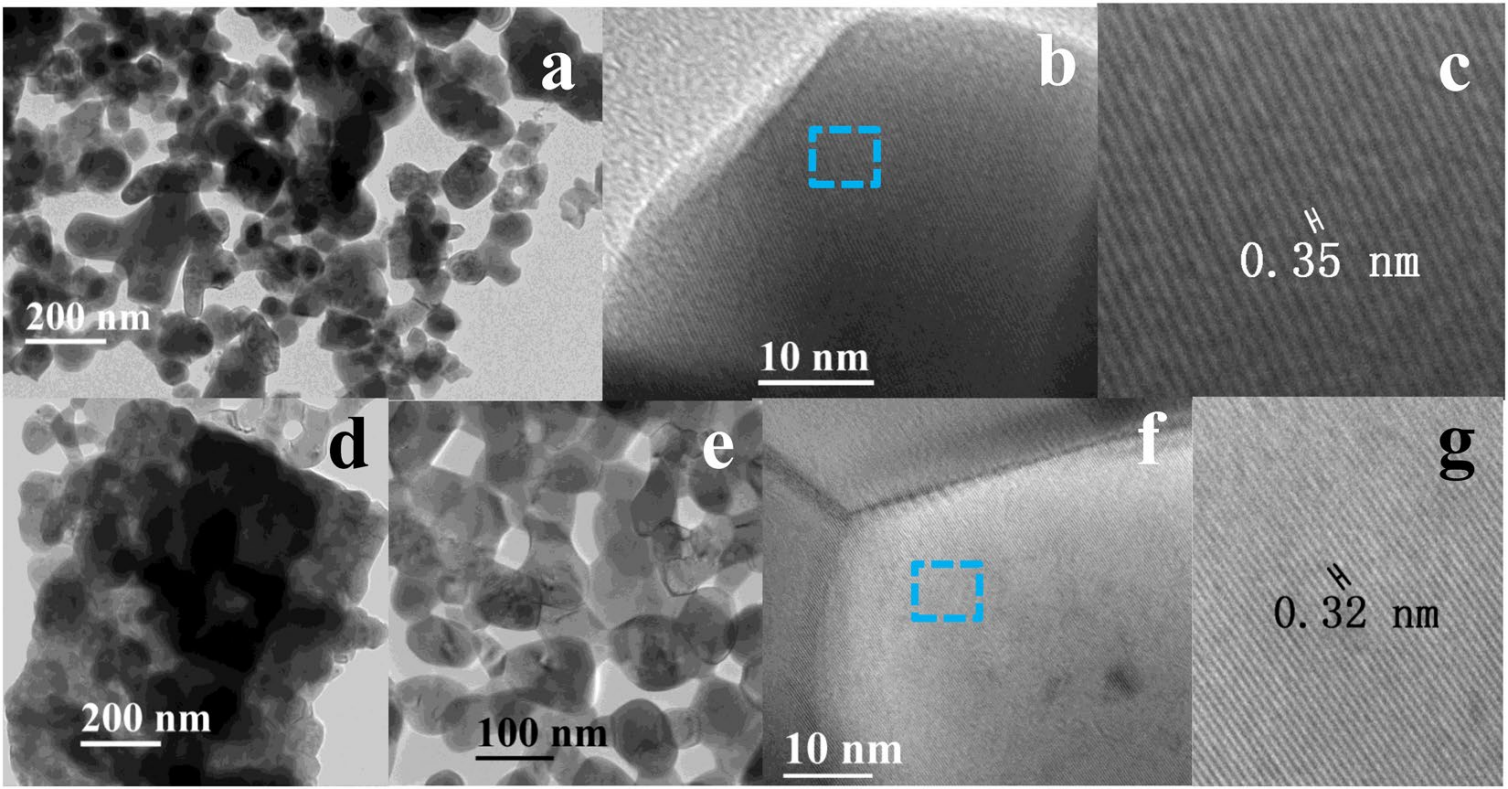
TEM images of as-synthesized ALC and ALS, (**a**) ALS, (**b**) high-resolution crystal lattice of ALS, (**c**) amplification of selected area in (**b**,**d** and **e**) ALC, (**f**) high-resolution crystal lattice of ALC, and (**g**) amplification of selected area in (**f**).

TEM provides insight into the morphology of the ALC and ALS as shown in Fig. 4. The images clearly show that the ALS (Fig. 4(a)) exhibit irregular bulk shape, which is consistent with the results of SEM. As shown in the image, the ALS consisted of many particles with the diameters as high as tens of nanometers. Fig. 4(d) shows the bulk structural characteristics of the ALC. Based on the image shown in Fig. 4(e), ALC is found to be comprised of a large number of interstitial Ag-LaFeO_3_ particles. There are many pores on the surface of the bulk with the pore size of approximately 50 nm, which is in accordance with the results observed in the high-magnification SEM image (Fig. 3(j), 400000x). High-magnification TEM images of the ALC and ALS are shown in Fig. 4(b,f) and the clearly observed the lattice stripes indicate good crystallinity. To obtain a clearer image of the lattice fringes, Fig. 4(c,g) shows a more distinct image of the selected areas. The fringe spacing of ALS and ALC are 0.35 nm and 0.32 nm, respectively.

**Figure 5:**
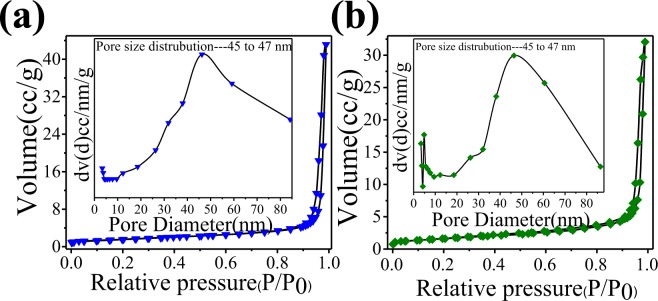
Isotherms of (**a**) ALS, and (**b**) ALC with pore size distribution curves shown in the insets.

The specific surface areas of the ALC and ALS mesoporous materials are determined by the N_2_ adsorption desorption isotherm curve and the distribution curves of the pore size are shown in Fig. 5. According to the International Union of Pure and Applied Chemistry, the H3 hysteresis loop indicates the pores are slit-like and formed by particle aggregation^29^. The ALS and ALC show obvious hysteresis loops at the P/P_0_ ranges of 0.6–1. Thus, the existence of pores is proven.

The BJH method (Fig. 5(a,b), inset) shows that ALS and ALC have porous structures. The size of the hole ranges from 2 nm to 90 nm, and the main peak is positioned at 45–47 nm. The S_BET_ values of the ALS and ALC were 5.66 m^2^ g^−1^ and 4.72 m^2^ g^−1^, respectively. The BET analysis indicated that ALS has a larger surface area, which means that ALS can provide sufficient surface for the gas reaction.

**Figure 6:**
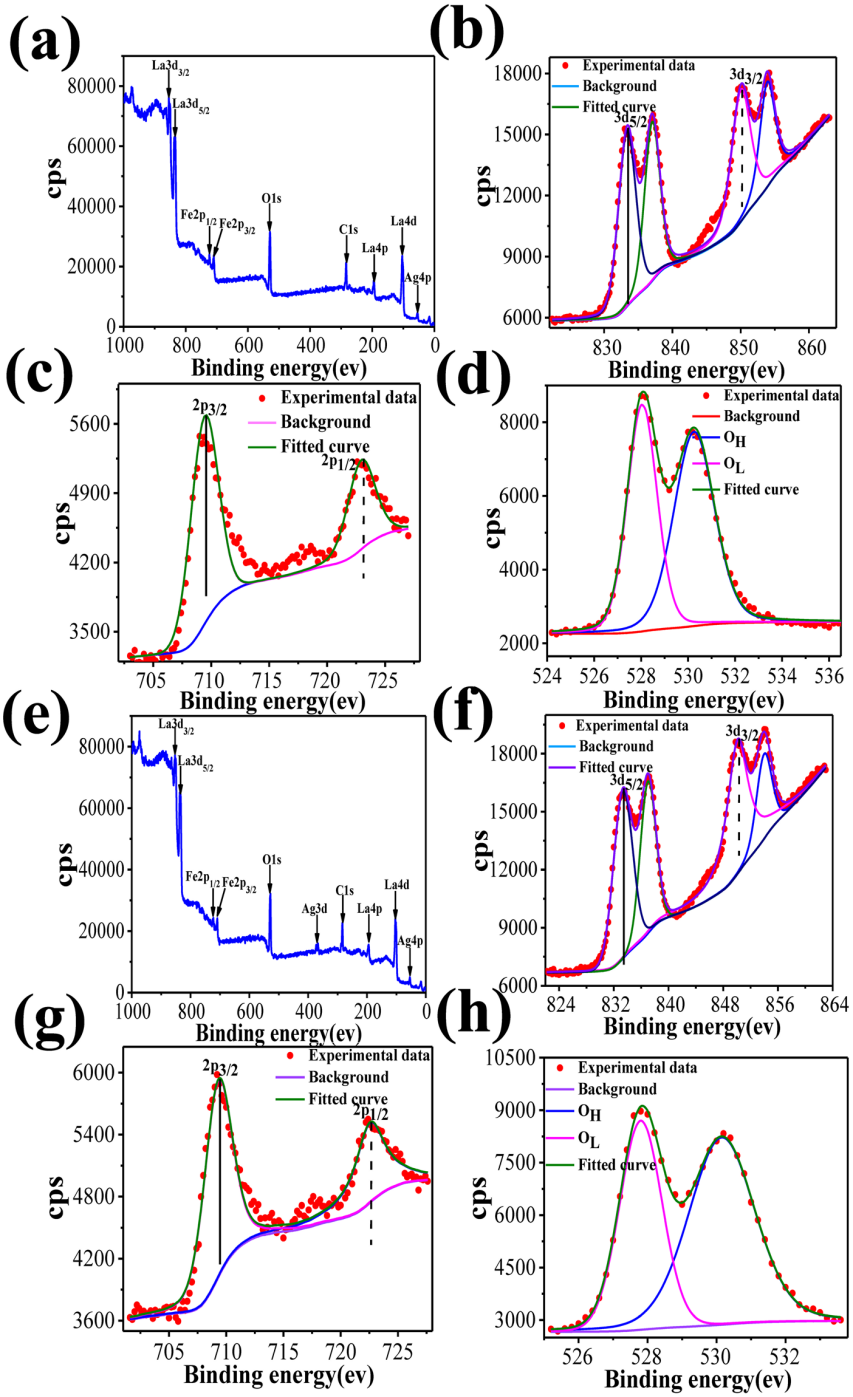
(**a**,**b**) XPS spectra of ALS and ALC, (**c**,**d**) high-magnification XPS spectra of La 3d for ALC and ALS, (**e**,**f**) high-magnification XPS spectrum of Fe 2p for ALC and ALS, respectively, and (**g**,**h**) high-magnification XPS spectra of O 1 s for ALC and ALS, respectively.

**Figure 7:**
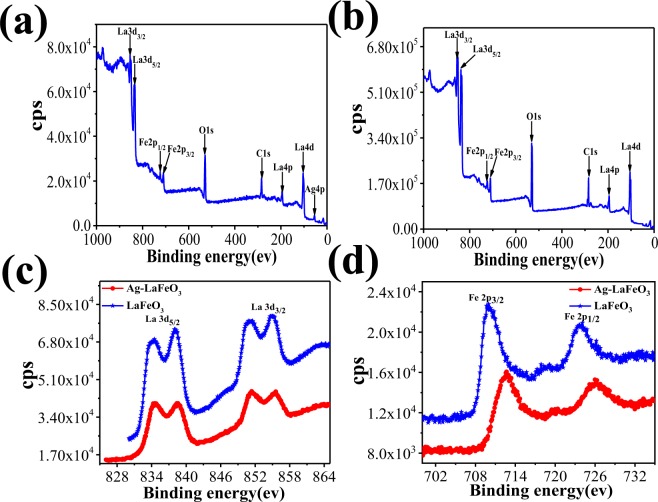
XPS full spectra of (**a**) Ag-LaFeO_3_ and (**b**) LaFeO_3_. (**c**) La 3d and (**d**) Fe 3p levels of Ag-LaFeO_3_ and LaFeO_3_.

The factors important for the gas-sensing mechanism include the surface control procedure, particle size, surface condition and adsorption and desorption of oxygen^30^. The characteristics of the gas sensor are affected by the surface states of the ALS and ALC. The XPS spectra of ALC and ALS were obtained in order to analyze the valence states of each element. Fig. 6(a,e) shows the XPS results for the ALS and ALC, with the standard peak of C1s at 284.8 eV. The spectra confirm the presence of Ag (3d, 4p), La (3d, 4p, 4d), Fe (2p), and O (1 s), indicating the high purity of the product. The XPS La 3d and Fe 2p energy levels display that the lanthanum ions and iron ions exist in the +3 state^31^. For the La 3d core level peaks (Fig. 6(b,f)), two peaks of lanthanum are seen from the peak; one is 3d_5/2_ of La^3+^ at 833.6 eV, and the other one is 3d_3/2_ of La^3+^ ions at 850.5 eV, which is due to the spin-orbit splitting of lanthanum oxide. Fig. 6(c,g) shows the XPS patterns of Fe 2p in ALS and ALC, and the two peaks of Fe 2p_3/2_ and Fe 2p_1/2_ were surveyed at 709.7 and 723.6 eV, respectively, which is due to the Fe^3+^ in the oxides^32^. The O 1 s XPS patterns (Fig. 6(d,h)) are wide as well as asymmetrical. At least two O chemical states are observed; one is O_L_ at 528.2 eV, and the other one is O_H_ at 530.5 eV, where O_L_ is attributed to lattice oxygen, and O_H_ is ascribed to the chemically adsorbed O^−^ or OH^−^. Therefore, the O 1 s XPS patterns fit two chemical conditions by Gauss’ rule^33^. The 528.2 eV peak of the O_L_ XPS spectrum is ascribed to the role of La-O and Fe-O of LaFeO_3_. The 530.5 eV peak of the O_H_ XPS is attributed to the OH^−^ resulting mainly from the chemically adsorbed water; this is due to lanthanum oxide, which can easily absorb moisture in the air. This metal-support interaction is a major element in the analysis of the mechanism of gas sensor. Figure 7(a,b) shows the XPS full spectra of Ag-LaFeO_3_ and LaFeO_3_, respectively. It can be seen from Fig. 7(c,d) that after the introduction of Ag, the binding energy corresponding to Fe and La shifted to a small direction. This shows that the doping of Ag is successful, but the amount of doping is too small to be detected. Therefore, the high magnification of Ag 3d is not available.

In order to evaluate the response of the gas sensor to methanol, the response of the prepared materials to 5 ppm methanol was detected at the operating temperatures in the 5–250 °C range. The gas response value reaches the maximum at the operating temperatures of 155 °C, 175 °C, 155 °C and 155 °C for ALSW, ALSM, ALCW and ALCM, respectively; as the operating temperature increases, the response decreases, as shown in Fig. 7(a–d). Therefore, 175 °C, 155 °C, 155 °C and 155 °C were used as the best operational temperatures of the ALSW, ALSM, ALCW and ALCM sensors, respectively.

**Figure 8:**
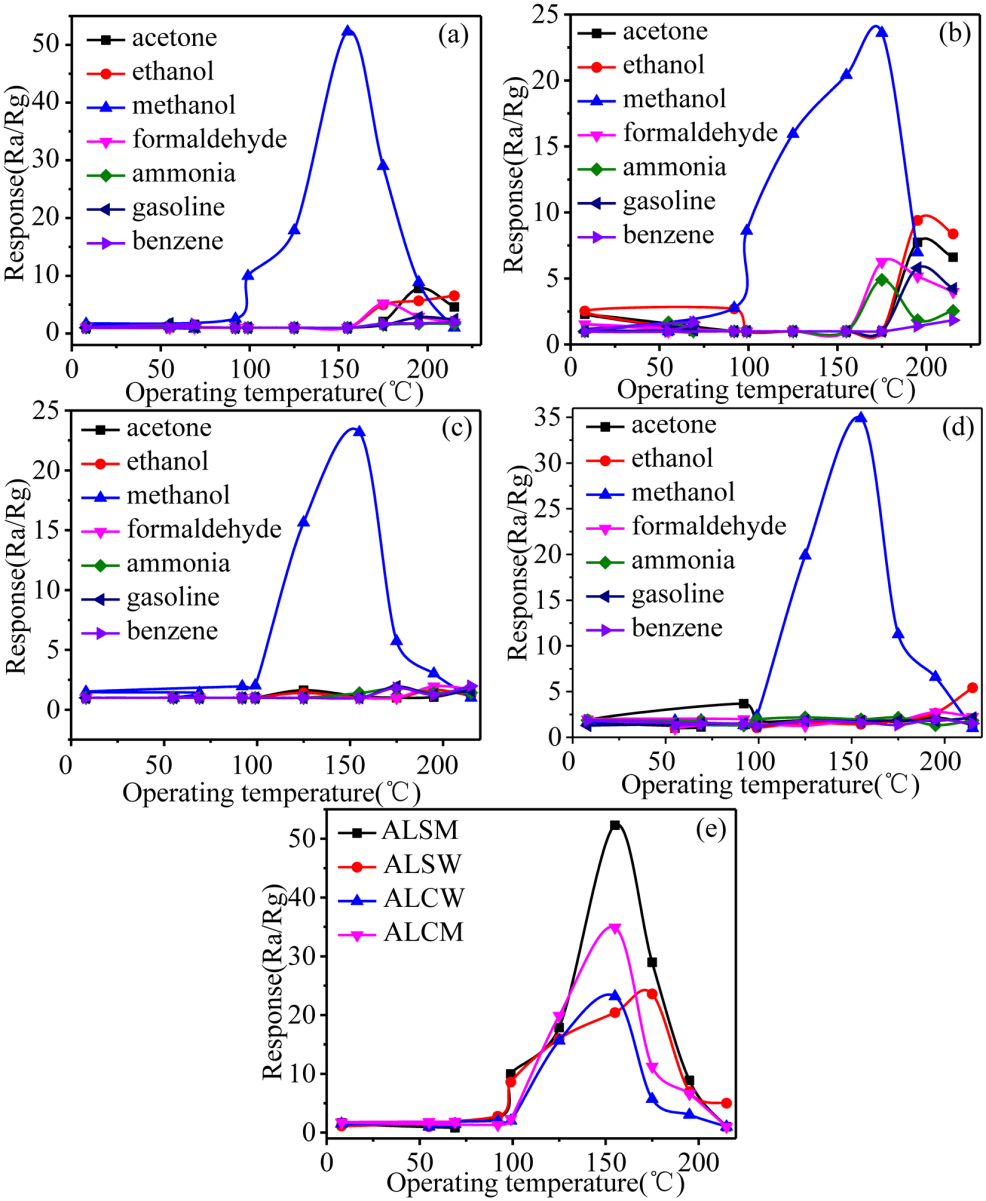
Response to different gases of the (**a**) ALSM, (**b**) ALSW, (**c**) ALCW and (**d**) ALCM, and (**e**) response of four devices to methanol.

Then, the response and selectivity of the four sensors were analyzed based on the data in Fig. 8. The responses of ALSM (52.29, Fig. 8(a)) and ALCM (34.89, Fig. 8(d)) are obviously superior to those of ALSW (23.6, Fig. 8(b)) and ALCW (23.2, Fig. 8(c)) for 5 ppm methanol at the best working temperature, compared to the reported values, and a good response is observed, as shown in Table 3 ^34–41^. All four sensors exhibit good selectivity to methanol. ALSM exhibits the best gas sensing properties among all devices (Fig. 8(e)) because the methanol molecules were introduced into the process of equipment preparation, which result in more methanol gas scattered on the surface of ALSM when ALSM was alternately placed in methanol gas and air. This will improve the gas response to methanol.

**Table 3:** Comparison of gas sensing properties of methanol sensors based on different materials.

Materials	Response	Detection limit (ppm)	References
Na:ZnO nanoflowers	1.8	500	^9^
Co_3_O_4_ nanosheets	225	1000	^34^
In_2_O_3_/SnO_2_	320.7	100	^35^
α-Fe_2_O_3_/Carbon nanotudes	12	100	^8^
In_2_O_3_/ZnO	15	100	^36^
NiO/ZnO	30	100	^37^
In_2_O_3_/Al_2_O_3_	23	100	^38^
α-Fe_2_O_3_	5	10	^39^
Porous TiO_2_	10	50	^40^
Zn_1−x_Cd_x_S	12	100	^41^
ALSW	23.6	5	This work
ALSM	52.29	5	This work
ALCW	23.2	5	This work
ALCM	34.89	5	This work

**Figure 9:**
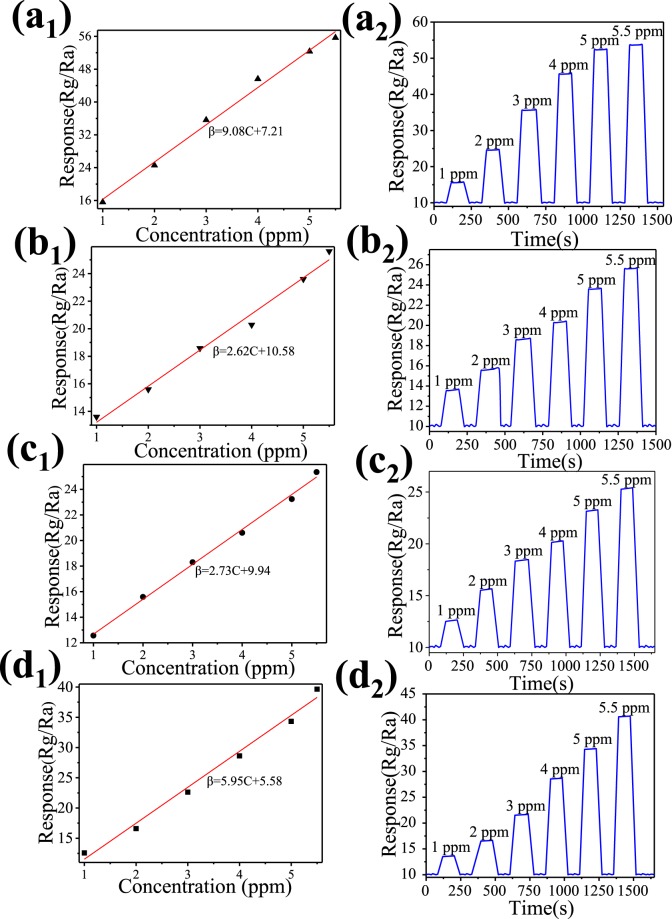
Response linearly increases with methanol concentration for (**a**) ALSM, (**b**) ALSW, (**c**) ALCW and (**d**) ALCM; dynamic response curves of (**e**) ALSM, (**f**) ALSW, (**g**) ALCW, and (**h**) ALCM.

Fig. 9 shows the relationships between the response and methanol concentration for the ALSM, ALSW, ALCW and ALCM in the methanol concentration range from 1 to 5.5 ppm. It can be seen in Fig. 9(a_1_–d_1_) that the responses increased linearly with the methanol concentration increasing from 1 to 5.5 ppm. The sensors can be used for real-time detection of methanol at the optimal working temperature. It can be found in Fig. 9(a_2_–d_2_) that the response of the sensor device exhibits a step-like distribution under different methanol concentrations. The response value dramatically increased with the increase in the methanol concentration. Furthermore, the response and recovery time are 32 s and 37 s, 34 s and 36 s, 37 s and 49 s, 36 s and 42 s.

For sensors based on metal oxides, several factors affect the gas sensing properties, such as surface area, porous structure and particle size. This further influences the spread and adsorption of the gases^42^. Methanol can interact with Ag-LaFeO_3_ in the preparation of the materials, which will further optimize the structure of ALSM. The structure of the materials was further optimized by introducing methanol into the device fabrication. This mechanism of the sensors ALSM and ALCM is defined as the excimer imprinting technique. Then, the mechanism of MIT was analyzed in detail^43–45^. ALSM and ALCM show better responses than ALSW and ALCW. This means that the preparation method of the device plays an important role in improving the response of the gas sensor. The gas sensing properties of the device are improved by the large surface area and multiple recognition sites of the gas sensing material^46,47^.

The working principle of the sensor is based on the change of the conductivity caused by the reaction between the gas molecule and the surface of the material. The porous structure of the material promotes the reaction of the gas and further improves the gas sensing property of the material. During the test process, the sensor was exposed to air at first, and oxygen adsorbed on the surface of LaFeO_3_ combined with the free electrons trapped on the LaFeO_3_ surface to form O_2_^**−**^, O^**−**^, O^2−^ and other oxygen ions^48^. The reactions are as follows:

1 $${{\rm{O}}}_{2({\rm{gas}})}\to {{\rm{O}}}_{2({\rm{ads}})}$$

2 $${{\rm{O}}}_{2({\rm{ads}})}+{{\rm{e}}}^{-}\to {{\rm{O}}}_{2({\rm{ads}})}^{-}$$

3 $${{\rm{O}}}_{2({\rm{ads}})}^{-}+{{\rm{e}}}^{-}\to 2{{\rm{O}}}_{({\rm{ads}})}^{-}$$

4 $${{\rm{O}}}_{({\rm{ads}})}^{-}+{{\rm{e}}}^{-}\to {{\rm{O}}}_{({\rm{ads}})}^{2-}$$

**Figure 10:**
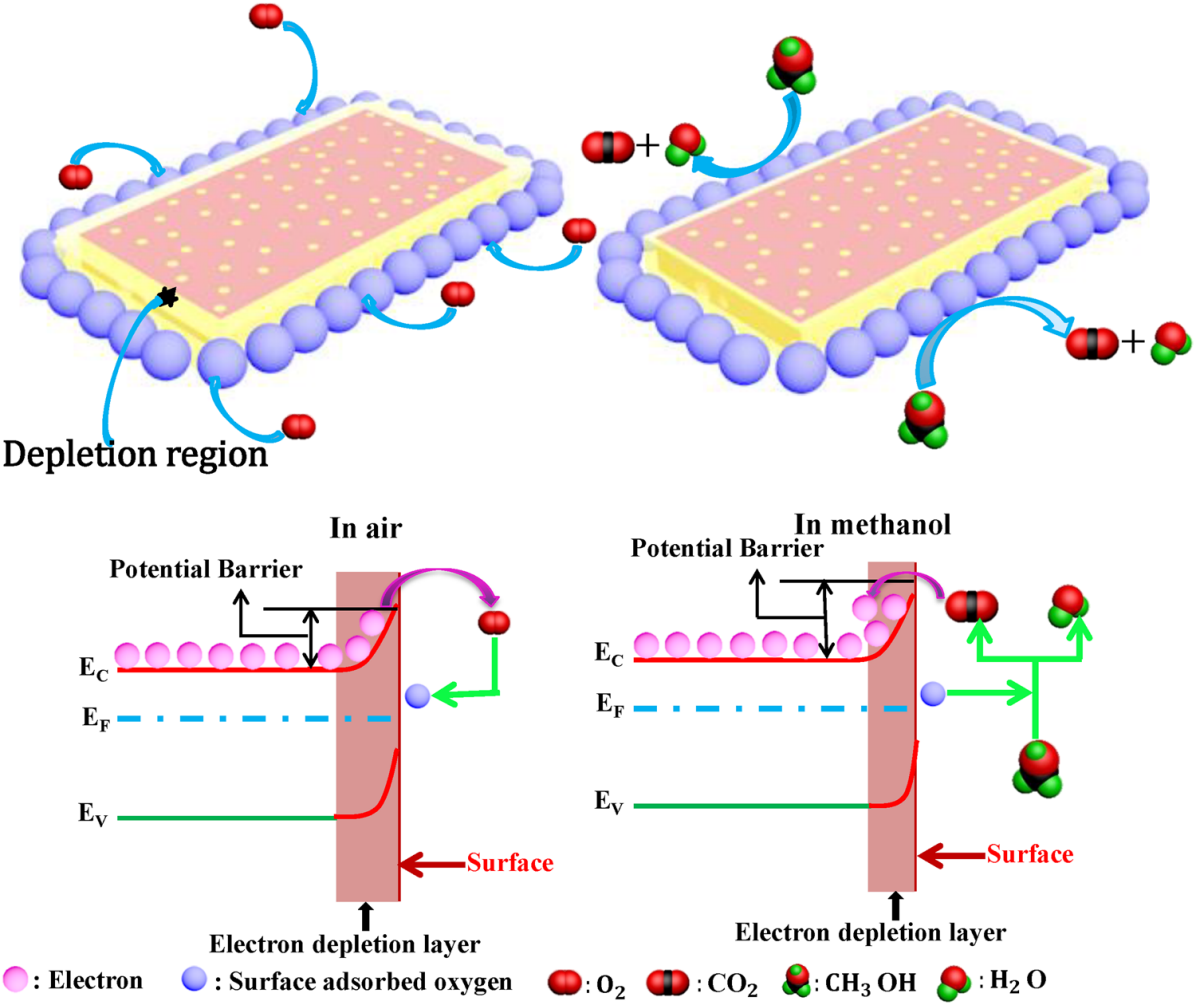
Mechanism of methanol sensor: (**a**) In air and (**b**) in methanol.

This process will form a thin space charge layer, and relatively low barriers are formed to lower the resistance^49^. When the material is in the presence of methanol (CH_3_OH), the reducing gases react with the adsorbed oxygen forming CO_2_ and H_2_O and releasing the electrons^50^. The space charge layer on the SLFO surface becomes thick, and the sensor resistance increases (Fig. 10). The reaction process is as follows:

5 $${{\rm{CH}}}_{3}{\rm{OH}}+2{{\rm{O}}}^{2-}\to {{\rm{CO}}}_{2}+{{\rm{H}}}_{2}{\rm{O}}+4{{\rm{e}}}^{-}$$

In summary, in this work, we applied quasi-MIT to design a methanol gas sensor. The experimental result indicates that the response and operating temperature of ALSM, ALSW, ALCW and ALCM are 52.29 and 155 °C, 23.60 and 175 °C, 23.20 and 155 °C, and 34.89 and 155 °C, respectively. The response and recovery time respectively are 32 s and 37 s, 34 s and 36 s, 37 s and 49 s, 36 s and 42 s. The maximum response to the other test gases is 8. The ALSM and ALCM sensors exhibit the most excellent responses. The design of a high-sensitivity methanol gas sensor based on quasi-MIT mechanism is reasonable. Furthermore, the pore number and specific surface area of ALSM are larger than those of ALCM, which accelerates the target gases to diffuse in or escape from the inner regions; therefore, ALSM displays a better gas response than ALCM. Our method is proven to be innovative for the design of a high-sensitivity methanol gas sensing device. This work provides important guidance for the design of high-response gas sensors.

## Methods

### Preparation of ALS

The chemical reagents used in the experiment were analytical reagents that were used without further treatment. The solvent is highly pure water (18 MΩ cm at 25 °C). In the prototype process, citric acid, lanthanum nitrate, iron nitrate, silver nitrate, PEG and deionized water were mixed under stirring at 90 °C for 10 hours as solution A. Then, solution A was placed in the microwave synthesizing device (CEM) for 4 hours at 75 °C. Methanol mixed with MAA was treated for 30 min by magnetic stirring and was let to stand to form solution B. Then, azobisisobutyronitrile (AIBN) was mixed with methanol, solution B and solution A. The final solution was treated by magnetic stirring at 50 °C for 12 h with a water bath and was then dried. Finally, the white powder of ALS was obtained after annealing at 800 °C for 2 h in an oven.

### Preparation of ALC

The ALC powder was synthesized via the combustion synthesis; here, citric acid, lanthanum nitrate, iron nitrate, silver nitrate, PEG and deionized water were mixed under stirring at 90 °C for 10 hours as solution D. The final mixture was treated for 30 min by magnetic stirring, and the prepared samples were oven-dried at 85 °C. The xerogel was heated at 300 °C for 30 min, and then sintering at 800 °C was carried out to obtain ALC.

### Device fabrication

The mesoporous material was fabricated via the sol-gel method (ALS) as well as combustion synthesis (ALC). The as-synthesized samples (ALS, ALC) were mixed with deionized water (ALSW and ALCW) and methanol (ALSM and ALCM) and ground to a mushy solution. For the detailed description of the fabrication method of the sensors, see ref.^51^.

### Characterization

X-ray diffraction (XRD) spectra were obtained using a Japan AXS D/MAX-3BX advanced device handled at 25 mA and 35 kV with Cu Kα scattered at 1.5406 Å wavelengths. The detailed morphology features and crystallization conditions of the material were surveyed via field emission scanning electron microscopy (FESEM, S-3400N) as well as transmission electron microscopy (JEM-2100, Hitachi, Japan). The functional group was identified by Fourier transform infrared spectroscopy (FT-IR, FTS-40). The BET surface area (S_BET_) of the material was measured using a Quadrasorb-evo instrument.
